# Morbidity and Length of Stay After Injury Among People Experiencing Homelessness in North America

**DOI:** 10.1001/jamanetworkopen.2024.0795

**Published:** 2024-02-28

**Authors:** Casey M. Silver, Arielle C. Thomas, Susheel Reddy, Shelbie Kirkendoll, Avery B. Nathens, Nabil Issa, Purvi P. Patel, Rebecca E. Plevin, Hemal K. Kanzaria, Anne M. Stey

**Affiliations:** 1 Department of Surgery, Northwestern University Feinberg School of Medicine, Chicago, Illinois; 2 Department of Surgery, Loyola University Medical Center, Maywood, Illinois; 3 American College of Surgeons, Chicago, Illinois; 4 Department of Surgery, Medical College of Wisconsin, Milwaukee; 5 Department of Surgery, University of Toronto, Toronto, Ontario, Canada; 6 Department of Surgery, University of California, San Francisco; 7 Department of Emergency Medicine, University of California, San Francisco

## Abstract

**Importance:**

Traumatic injury is a leading cause of hospitalization among people experiencing homelessness. However, hospital course among this population is unknown.

**Objective:**

To evaluate whether homelessness was associated with increased morbidity and length of stay (LOS) after hospitalization for traumatic injury and whether associations between homelessness and LOS were moderated by age and/or Injury Severity Score (ISS).

**Design, Setting, and Participants:**

This retrospective cohort study of the American College of Surgeons Trauma Quality Programs (TQP) included patients 18 years or older who were hospitalized after an injury and discharged alive from 787 hospitals in North America from January 1, 2017, to December 31, 2018. People experiencing homelessness were propensity matched to housed patients for hospital, sex, insurance type, comorbidity, injury mechanism type, injury body region, and Glasgow Coma Scale score. Data were analyzed from February 1, 2022, to May 31, 2023.

**Exposures:**

People experiencing homelessness were identified using the TQP’s alternate home residence variable.

**Main Outcomes and Measures:**

Morbidity, hemorrhage control surgery, and intensive care unit (ICU) admission were assessed. Associations between homelessness and LOS (in days) were tested with hierarchical multivariable negative bionomial regression. Moderation effects of age and ISS on the association between homelessness and LOS were evaluated with interaction terms.

**Results:**

Of 1 441 982 patients (mean [SD] age, 55.1 [21.1] years; (822 491 [57.0%] men, 619 337 [43.0%] women, and 154 [0.01%] missing), 9065 (0.6%) were people experiencing homelessness. Unmatched people experiencing homelessness demonstrated higher rates of morbidity (221 [2.4%] vs 25 134 [1.8%]; *P* < .001), hemorrhage control surgery (289 [3.2%] vs 20 331 [1.4%]; *P* < .001), and ICU admission (2353 [26.0%] vs 307 714 [21.5%]; *P* < .001) compared with housed patients. The matched cohort comprised 8665 pairs at 378 hospitals. Differences in rates of morbidity, hemorrhage control surgery, and ICU admission between people experiencing homelessness and matched housed patients were not statistically significant. The median unadjusted LOS was 5 (IQR, 3-10) days among people experiencing homelessness and 4 (IQR, 2-8) days among matched housed patients (*P* < .001). People experiencing homelessness experienced a 22.1% longer adjusted LOS (incident rate ratio [IRR], 1.22 [95% CI, 1.19-1.25]). The greatest increase in adjusted LOS was observed among people experiencing homelessness who were 65 years or older (IRR, 1.42 [95% CI, 1.32-1.54]). People experiencing homelessness with minor injury (ISS, 1-8) had the greatest relative increase in adjusted LOS (IRR, 1.30 [95% CI, 1.25-1.35]) compared with people experiencing homelessness with severe injury (ISS ≥16; IRR, 1.14 [95% CI, 1.09-1.20]).

**Conclusions and Relevance:**

The findings of this cohort study suggest that challenges in providing safe discharge to people experiencing homelessness after injury may lead to prolonged LOS. These findings underscore the need to reduce disparities in trauma outcomes and improve hospital resource use among people experiencing homelessness.

## Key Points

**Question:**

Do people experiencing homelessness have increased morbidity and length of hospital stay after injury compared with housed patients?

**Findings:**

In this cohort study of 1 441 982 patient encounters from the American College of Surgeons Trauma Quality Programs, people experiencing homelessness demonstrated similar rates of morbidity compared with propensity score–matched housed patients, but with a longer adjusted length of stay (LOS). The association between homelessness and increased LOS was greatest among those 65 years and older and with minor injury.

**Meaning:**

These findings underscore the challenges in providing safe hospital discharge for people experiencing homelessness after injury, leading to prolonged LOS.

## Teaser

This cohort study evaluates the association between homelessness and increased morbidity and length of stay after hospitalization for traumatic injury and the extent to which this association is moderated by age and/or Injury Severity Score.

## Introduction

Approximately 580 000 people experienced homelessness on any given day in 2020 across the US, and this incidence has increased since the start of the COVID-19 pandemic.^1^ People experiencing homelessness have worse health-related quality of life than housed individuals.^2,3,4,5^ Structural barriers to accessing health care for people experiencing homelessness lead to high rates of emergency department use and hospitalization, resulting in significant health care costs.^6,7,8,9,10^

Traumatic injury is the second leading reason for hospitalization among people experiencing homelessness.^11^ A previous study^9^ demonstrated that injured people experiencing homelessness are more likely to be admitted to the hospital than housed individuals. However, little is known about their subsequent hospital course and the incidence of morbidity, surgical procedures, and intensive care unit (ICU) admissions.^3^ Length of stay (LOS) is a measure of resource use that closely approximates total cost of care.^12^ Prolonged LOS impairs bed turnover and can lead to decreased capacity and strain in the emergency department and wards. Increased morbidity and LOS among people experiencing homelessness admitted for physical and behavioral health complaints have been documented.^10,13^ However, injury admissions differ from other admissions because injury often causes new physical disabilities, wounds, and pain. Furthermore, many studies were limited to single centers, limiting generalizability.^14^ Hospital course among injured people experiencing homelessness has not been studied nationally. Defining which patients have the greatest increase in LOS can help tailor discharge planning efforts.

This cohort study sought to evaluate hospital course after injury among people experiencing homelessness compared with housed patients in North America. Our objectives were to (1) assess the incidence of morbidity, surgical intervention, and ICU admission in people experiencing homelessness compared with housed patients; (2) investigate associations between homelessness and LOS; and (3) evaluate whether age and injury severity modified the association.

## Methods

The Northwestern University Institutional Review Board approved this cohort study and waived the need for informed consent owing to the use of deidentified data. The study followed the Strengthening the Reporting of Observational Studies in Epidemiology (STROBE) reporting guideline.

### Data Source

We conducted a retrospective cohort study of the American College of Surgeons (ACS) Trauma Quality Programs (TQP) data. The ACS TQP includes data from ACS Trauma Quality Improvement Program–participating centers as well as a small number of non–ACS-verified centers that adhere to the National Trauma Data Standard. Together, these data represent an incident-based injury registry of over 7.5 million records. Hospitals participating in the TQP are located across the US and Canada. They are mostly ACS-verified level I or level II trauma centers or regionally designated trauma centers. Dedicated, trained abstractors recorded patient demographic, clinical, injury, and hospital data.^15^

### Study Population

We identified encounters of patients 18 years or older admitted to a participating hospital following injury from January 1, 2017, to December 31, 2018. We excluded patients who died in the hospital (n = 50 645 [308 (0.6%) experiencing homelessness]), as death is a known confounder of LOS, patients who left against medical advice (n = 40 577 [1390 (3.4%) experiencing homelessness]), and patients transferred to another hospital for inpatient care (n = 19 142 [115 (0.6%) experiencing homelessness]).^16^ Finally, we excluded encounters with missing LOS data (n = 10 644 [35 (0.3%) experiencing homelessness]) (Figure).

**Figure.**
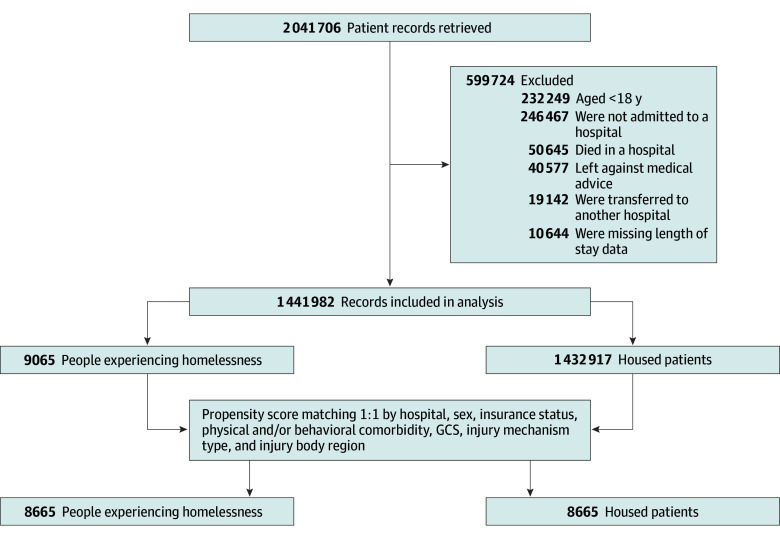
Patient Selection Schema Patient selection was based on American College of Surgeons Trauma Quality Programs data. GCS indicates Glasgow Coma Scale.

We defined a subcohort to address a priori known differences in demographic, clinical, and injury characteristics between people experiencing homelessness and housed patients. Many of these characteristics are also associated with increased morbidity and LOS.^17^ This subcohort consisted of people experiencing homelessness matched by propensity score to housed patients as described below.

### Exposure

Our exposure of interest was homelessness. People experiencing homelessness were identified using TQP’s alternate home residence variable (including homeless individuals, undocumented citizens, migrant workers). The TQP abstractors recorded this variable when patients did not have a temporary or permanent residence zip code listed on presentation to the emergency department. We classified those listed as homeless as people experiencing homelessness, while undocumented citizens and migrant workers were considered housed. The alternate home residence variable was not completed when a patient had a residence zip code listed; we labeled these records as housed. This variable has been used in another study of injured people experiencing homelessness by Silver et al.^9^

### Outcomes of Interest

Our primary outcome was LOS (in days). We evaluated LOS in 2 ways. First, we evaluated LOS as a continuous, nonnormally distributed outcome variable. Second, we evaluated LOS as a binary variable of whether or not LOS was greater than 30 days.

Our secondary outcomes were morbidity, hemorrhage control surgery, and ICU admission. We created a composite variable of any morbidity that included acute kidney injury, acute respiratory distress syndrome, cardiac arrest with cardiopulmonary resuscitation, venous thromboembolism, myocardial infarction, severe sepsis, surgical site infection, and unplanned intubation. These outcomes are included in morbidity measures used to assess surgical quality by the ACS^18^ and/or the Agency for Healthcare Research and Quality.^19^ Hemorrhage control surgery, as defined by TQP, includes laparotomy, thoracotomy, sternotomy, extremity surgery, neck surgery, traumatic amputation, other skin or soft tissue procedures, and extraperitoneal pelvic packing.^20^ We considered patients to have had surgery if they underwent 1 or more of these procedures. We evaluated ICU admission at any point during the patient’s hospital course.

### Covariates

Demographic variables in TQP included sex, race and ethnicity, and insurance type (private, Medicaid, Medicare, self-pay, or other). Race and ethnicity data were based on self-report or report of a family member and were characterized using separate race and ethnicity variables per National Trauma Data Bank definitions.^15^ Categories included Hispanic, non-Hispanic Black, non-Hispanic White, and other race or ethnicity (including American Indian and Alaska Native, Asian, and Native Hawaiian or Other Pacific Islander, grouped due to low frequency among people experiencing homelessness). Racial and ethnic disparities in outcomes and hospital use have been demonstrated, and thus we included them as a proxy for systemic racism.^21^ Physical health comorbidities captured by TQP included standard Elixhauser comorbidities such as heart disease, chronic obstructive pulmonary disease, diabetes, hypertension, liver disease, and malignant neoplasms.^22^ Behavioral health comorbidities included bipolar disorder, schizophrenia, major depressive disorder, posttraumatic stress disorder, and antisocial personality disorder. Included injury characteristics were injury mechanism type (blunt, penetrating, or other), injury body region, and Glasgow Coma Scale score.^23,24^ We used *International and Statistical Classification of Diseases and Related Health Problems, Tenth Revision* diagnosis codes to classify injury body region according to the Centers for Disease Control and Prevention Injury Mortality Diagnosis Matrix.^25^ Missing patient-level data were not imputed.

Hospitals’ trauma center status was defined by ACS verification level or, for those not ACS verified, state designation. When hospital-level data were known for some encounters and missing for others with the same hospital identification, missing hospital data were imputed under the assumption that hospital data would be the same for all patients admitted to a given hospital (<1% of encounters).

### Moderation Analysis

We assessed moderation effects of age and Injury Severity Score (ISS) on the association between homelessness and LOS. These variables were selected due to increasing age of the US homeless population and prior studies demonstrating differential associations between homelessness and hospital admission by ISS.^9,26^ Age was considered categorically (18-35, 36-50, 51-64, and ≥65 years), given the highly skewed distribution. The ISS was also considered categorically using standard categories for minor (1-8), moderate (9-15), and severe (≥16) injury.^23^

### Propensity Score Matching

We used propensity score matching to reduce confounding given known differences between people experiencing homelessness and housed patients.^27^ We used the Stata package psmatch2 (StataCorp LLC) to create a propensity score model estimating the probability of experiencing homelessness. This model matched people experiencing homelessness to housed patients on sex, insurance, physical and/or behavioral comorbidity, injury mechanism type, Glasgow Coma Scale score, and injury body region. Hospital identification was used as an exact-matching criterion to account for differences between hospitals. Propensity matching was performed using a greedy 1:1 nearest-neighbor algorithm with a specified maximum caliper width of 0.5.^28^ Postmatch comparisons with standardized differences demonstrated a near complete match of people experiencing homelessness with better balanced distributions for demographic, clinical, and injury characteristics, though differences in age distributions persisted (eTable 1 in Supplement 1). A standardized difference less than 0.20 was considered sufficient balance and less than 0.10 was considered ideal.^21,29^

### Statistical Analysis

In the unmatched cohort, we compared demographic, clinical, hospital, and injury characteristics between people experiencing homelessness and housed patients using χ^2^ tests of independence. Unadjusted rates of morbidity, hemorrhage control surgery, and ICU admission were compared using χ^2^ tests of independence. Unadjusted LOS was compared between people experiencing homelessness and housed patients using Mann-Whitney tests. Rates of LOS longer than 30 days were calculated and compared with χ^2^ tests of independence.

After matching, rates of morbidity, hemorrhage control surgery, and ICU admission were compared between people experiencing homelessness and matched housed patients using McNemar tests. Unadjusted LOS was compared between matched people experiencing homelessness and housed patients using the Wilcoxon signed rank test. Rates of LOS longer than 30 days were compared with McNemar tests. All tests were 2 sided with α < .05 considered statistically significant.

In the matched cohort, we estimated hierarchical multivariable negative binomial regression to evaluate associations between homelessness and adjusted LOS. We used hierarchical multivariable logistic regression to evaluate the association between homelessness and odds of adjusted LOS longer than 30 days. Models included random effects at the level of both propensity score–matched pairs and hospitals while controlling for age, ISS, morbidity, hemorrhage control surgery, and ICU admission.

We created interaction terms to evaluate whether age and/or injury severity moderated the association between homelessness and LOS. These interaction terms were included in 2 separate additional hierarchical multivariable negative binomial regression models. The first interaction term was homelessness × categorical age. The second interaction term was homelessness × categorical ISS. These interaction terms tested whether the association between homelessness and LOS was strengthened or weakened by the moderator variable. We clustered observations by both propensity score–matched pairs and hospital identification while controlling for age, ISS, any morbidity, hemorrhage control surgery, and ICU admission. Data were analyzed from February 1, 2022, to May 31, 2023. Analyses were performed using Stata MP, version 17.0 (StataCorp LLC).

## Results

### Unmatched Analysis

There were 1 441 982 patient encounters within 787 hospitals (822 491 [57.0%] men, 619 337 [43.0%] women, and 154 [0.01%] missing; mean [SD] age, 55.1 [21.1] years). In terms of race and ethnicity, 78 057 participants (5.4%) were Hispanic, 175 868 (12.2%) were non-Hispanic Black, 985 782 (68.4%) were non-Hispanic White, and 143 676 (10.0%) were of other race or ethnicity. People experiencing homelessness constituted 9065 (0.6%) encounters. People experiencing homelessness were less frequently older than 65 years than housed patients (583 [6.4%] vs 512 246 [33.7%]; *P* < .001) and more often uninsured (1970 [21.7%] vs 135 744 [9.5%]; *P* < .001) or insured by Medicaid (4533 [50.0%] vs 188 149 [13.1%]; *P* < .001). People experiencing homelessness more often presented with severe injury (ISS ≥16, 1802 [19.9%] vs 222 479 [15.5%]; *P* < .001) compared with housed patients (Table 1).

**Table 1.** Characteristics of Unmatched Cohort of People Experiencing Homelessness and Housed Patients in the Trauma Quality Programs, 2017 to 2018

Characteristic	Cohort^a^		*P* value^b^
	People experiencing homelessness (n = 9065 [0.6%])	Housed patients (n = 1 432 917 [99.4%])	
Sex			
Men	7642 (84.3)	814 849 (56.9)	<.001
Women	1422 (15.7)	617 915 (43.1)	
Missing	1 (0.01)	153 (0.01)	
Age, y			
18-35	2408 (26.6)	322 721 (22.5)	<.001
36-50	2817 (31.1)	216 151 (15.1)	
51-64	3235 (35.7)	273 012 (19.1)	
≥65	583 (6.4)	512 246 (35.7)	
Missing	22 (0.2)	108 787 (7.6)	
Race and ethnicity			
Hispanic	918 (10.1)	77 139 (5.4)	<.001
Non-Hispanic Black	1917 (21.1)	173 951 (12.1)	
Non-Hispanic White	4582 (50.5)	981 200 (68.5)	
Other^c^	1416 (15.6)	142 260 (9.9)	
Missing	232 (2.6)	58 367 (4.1)	
Insurance			
Private	1051 (11.6)	466 295 (32.5)	<.001
Uninsured	1970 (21.7)	135 744 (9.5)	
Medicaid	4533 (50.0)	188 149 (13.1)	
Medicare	930 (10.3)	539 216 (37.6)	
Other	545 (6.0)	76 919 (5.4)	
Missing	36 (0.4)	26 594 (1.9)	
Comorbidities			
Any physical comorbidity	3280 (36.2)	819 800 (57.2)	<.001
Any behavioral comorbidity	2083 (23.0)	162 837 (11.4)	<.001
Hospital characteristics			
Trauma center			
Level I	5716 (63.1)	746 031 (52.1)	<.001
Level II	2972 (32.8)	524 728 (36.6)	
Nontrauma	345 (3.8)	149 734 (10.4)	
Missing	32 (0.4)	12 424 (0.9)	
Injury characteristics			
Injury mechanism type			
Blunt	6922 (76.4)	1 283 574 (89.6)	<.001
Penetrating	1520 (16.8)	102 662 (7.2)	
Unknown or other	88 (1.0)	15 225 (1.1)	
Missing	535 (5.9)	31 456 (2.2)	
Injury body region^d^			
Head or neck	5784 (63.8)	659 366 (46.0)	<.001
Spine	1506 (16.6)	268 831 (18.8)	<.001
Torso	3676 (40.6)	503 416 (35.1)	<.001
Extremity	5024 (55.4)	892 774 (62.3)	<.001
ISS			
Mild (1-8)	4253 (46.9)	641 161 (44.7)	<.001
Moderate (9-15)	2998 (33.1)	566 954 (39.6)	
Severe ≥16	1802 (19.9)	222 479 (15.5)	
Missing	12 (0.1)	2323 (0.2)	
Initial GCS score^e^			
3-8	561 (6.2)	48 371 (3.4)	<.001
9-12	501 (5.5)	29 136 (2.0)	
13-15	7654 (84.4)	1 273 869 (88.9)	
Missing	349 (3.8)	81 541 (5.7)	

Abbreviations: GCS, Glasgow Coma Scale; ISS, Injury Severity Score.

^a^ Data are expressed as No. (%) of cohort individuals. Percentages have been rounded and may not total 100.

^b^ Derived from χ^2^ tests of independence.

^c^ Includes American Indian or Alaska Native, Asian, and Native Hawaiian or Other Pacific Islander.

^d^ Patients may present with injury to more than 1 body region.

^e^ Higher scores indicate better response.

In the unmatched cohort, unadjusted rates of any morbidity were higher among people experiencing homelessness (221 [2.4%] vs 25 134 [1.8%]; *P* < .001) compared with housed patients. People experiencing homelessness also demonstrated higher rates of hemorrhage control surgery (289 [3.2%] vs 20 331 [1.4%]; *P* < .001) and ICU admission (2353 [26.0%] vs 307 714 [21.5%]; *P* < .001) (Table 2). Unadjusted median LOS among people experiencing homelessness was 5 (IQR, 3-9) days compared with 4 (IQR, 3-7) days among housed patients (*P* < .001). People experiencing homelessness demonstrated higher rates of LOS longer than 30 days (949 [10.5%] vs 23 330 [1.6%]; *P* < .001).

**Table 2.** Unadjusted Hospital Events in Unmatched Cohort of People Experiencing Homelessness and Housed Patients in the Trauma Quality Programs, 2017 to 2018

Hospital event	Cohort^a^		*P* value^b^
	People experiencing homelessness (n = 9065 [0.6%])	Housed patients (n = 1 432 917 [99.4%])	
Morbidity			
Acute kidney injury	37 (0.4)	5367 (0.4)	.60
Acute respiratory distress syndrome	19 (0.2)	2920 (0.2)	.90
Cardiac arrest with CPR	26 (0.3)	2765 (0.2)	.04
Venous thromboembolism	29 (0.3)	4199 (0.3)	.64
Myocardial infarction	5 (0.1)	1938 (0.1)	.04
Severe sepsis	29 (0.3)	2848 (0.2)	.01
Surgical site infection	25 (0.3)	1268 (0.1)	<.001
Unplanned intubation	113 (1.2)	10 852 (0.8)	<.001
Any morbidity	221 (2.4)	25 134 (1.8)	<.001
Hemorrhage control surgery	289 (3.2)	20 331 (1.4)	<.001
ICU admission	2353 (26.0)	307 714 (21.5)	<.001
LOS, median (IQR), d (continuous)	5 (3-9)	4 (3-7)	<.001
LOS, mean (SD), d (continuous)	9.4 (15.2)	6.3 (9.3)	<.001
LOS >30 d	494 (10.5)	23 330 (1.6)	<.001

Abbreviations: CPR, cardiopulmonary resuscitation; ICU, intensive care unit; LOS, length of stay.

^a^ Unless otherwise indicated, data are expressed as No. (%) of cohort individuals.

^b^ Derived from χ^2^ tests of independence.

### Matched Analysis

The propensity score–matched cohort consisted of 8665 pairs admitted to 378 hospitals. Matched people experiencing homelessness had a trend toward increased rates of surgical site infection (25 [0.3%] vs 11 [0.1%]; *P* = .02) and unplanned intubation (109 [1.3%] vs 78 [0.9%]; *P* = .02) compared with housed patients. However, differences in rates of any morbidity, hemorrhage control surgery, and ICU admission were not statistically significant (eTable 2 in Supplement 1).

Matched people experiencing homelessness demonstrated higher unadjusted median LOS than housed patients (5 [IQR, 3-10] vs 4 [IQR, 2-8] days; *P* < .001) as well as higher rates of LOS longer than 30 days (478 [5.5%] vs 327 [3.8%]; *P* < .001). On multivariable analysis, people experiencing homelessness had an associated 22.1% longer LOS (incident rate ratio [IRR], 1.22 [95% CI, 1.19-1.25]; *P* < .001) (Table 3) when controlling for age, ISS, any morbidity, hemorrhage control surgery, and ICU admission. People experiencing homelessness also demonstrated significantly higher adjusted odds of LOS longer than 30 days (odds ratio, 1.59 [95% CI, 1.33-1.89]; *P* < .001) (eTable 3 in Supplement 1).

**Table 3.** Multivariable Model for Length of Stay in 8665 Matched Pairs of People Experiencing Homelessness and Housed Patients in the Trauma Quality Programs, 2017 to 2018

Characteristic	IRR (95% CI)	*P* value^a^
Housing status		
Housed	1 [Reference]	NA
Experiencing homelessness	1.22 (1.19-1.25)	<.001
Age, y		
18-35	1 [Reference]	NA
36-50	1.12 (1.09-1.16)	<.001
51-64	1.26 (1.21-1.29)	<.001
≥65	1.35 (1.29-1.42)	<.001
ISS^b^		
1-8	1 [Reference]	NA
9-15	1.41 (1.37-1.45)	<.001
≥16	2.40 (2.32-2.49)	<.001
Any morbidity		
No	1 [Reference]	NA
Yes	2.81 (2.62-3.01)	<.001
Hemorrhage control surgery		
No	1 [Reference]	NA
Yes	1.67 (1.57-1.78)	<.001
ICU admission		
No	1 [Reference]	NA
Yes	1.36 (1.32-1.40)	<.001

Abbreviations: ICU, intensive care unit; IRR, incident rate ratio; ISS, Injury Severity Score; NA, not applicable.

^a^ Estimated from hierarchical negative binomial regression models allowing for random effects at the pair level and at the hospital level.

^b^ Higher scores indicate greater severity of injury.

Interaction analyses assessed for moderation effects of age and ISS. The difference in unadjusted median LOS between people experiencing homelessness and housed patients was 1 day across all age groups (Table 4). However, the direct association between homelessness and adjusted LOS increased with advancing age. People experiencing homelessness aged 18 to 35 years demonstrated a 16.3% increased adjusted LOS compared with housed patients (IRR, 1.16 [95% CI, 1.12-1.21]; *P* < .001), while people experiencing homelessness who were 65 years or older had an associated 42.4% increased adjusted LOS (IRR, 1.42 [95% CI, 1.32-1.54]; *P* < .001) (Table 4). This moderation of age on the direct association between homelessness and adjusted LOS was statistically significant (*P* < .001).

**Table 4.** Moderation of the Association Between Housing Status and Length of Stay by Age and ISS in People Experiencing Homelessness and Matched Housed Patients in the Trauma Quality Programs, 2017 to 2018

Characteristic	Unadjusted LOS, median (IQR), d		Adjusted IRR (95% CI)^a^	Interaction term *P* value^b^
	People experiencing homelessness	Housed patients		
Age, y				
18-35	4 (3-8)	3 (2-7)	1.16 (1.12-1.21)	NA
36-50	5 (3-8)	4 (2-7)	1.17 (1.12-1.23)	.74
51-64	6 (3-11)	5 (3-8)	1.28 (1.22-1.33)	.003
≥65	6 (4-10)	5 (3-9)	1.42 (1.32-1.54)	<.001
ISS^c^				
≥16	13 (7-25)	12 (7-25)	1.14 (1.09-1.20)	NA
9-15	6 (4-10)	5 (3-7)	1.19 (1.14-1.23)	.24
1-8	4 (2-6)	3 (2-4)	1.30 (1.25-1.35)	<.001

Abbreviations: IRR, adjusted incident rate ratio; ISS, Injury Severity Score; LOS, length of stay; NA, not applicable.

^a^ Derived from hierarchical multivariable negative binomial regression models controlling for age, ISS, any morbidity, hemorrhage control surgery, and ICU admission. Observations are clustered at the level of the matched pair and at the level of the hospital.

^b^ Calculated as the interaction term between the variable of interest (age and ISS) and experiencing homelessness. Interactions between age and ISS were modeled separately. Value NA indicates reference category.

^c^ Higher scores indicate greater severity of injury.

The difference in unadjusted median LOS between people experiencing homelessness and housed patients was 1 day across all ISS categories (Table 4). However, on multivariable analysis, the direct association between homelessness and LOS was greatest for those with an ISS of 1 to 8 (minor injury), with a 30.0% increased adjusted LOS (IRR, 1.30 [95% CI, 1.25-1.35]), while people experiencing homelessness with an ISS of 16 or greater had an associated 14.4% increased adjusted LOS (IRR, 1.14 [95% CI, 1.09-1.20]) (Table 4). This moderation of ISS on the direct association between homelessness and adjusted LOS was statistically significant (*P* < .001).

## Discussion

Traumatic injury is the second-leading cause of hospitalization and a common cause of death for people experiencing homelessness.^11,30^ The hospital course among people experiencing homelessness remains poorly understood despite increasing recognition of housing as a health-related social need.^2^ To our knowledge, this study constitutes the first evaluation of hospital course and LOS in North America among people experiencing homelessness who are discharged alive following traumatic injury. We found that unmatched people experiencing homelessness had higher unadjusted rates of morbidity, hemorrhage control surgery, and ICU admission compared with housed patients, though these differences did not persist in the matched cohort. Nonetheless, matched people experiencing homelessness had an associated 22.1% increased adjusted LOS compared with housed patients. The relative increase in adjusted LOS was greatest among people experiencing homelessness who were 65 years or older and among those with minor injury.

Few studies have examined morbidity and incidence of surgery among injured people experiencing homelessness. A study by Decker et al^31^ found that people experiencing homelessness and undergoing emergent surgery had comparable rates of in-hospital mortality and postoperative complications compared with housed patients. However, their study did not include patients with traumatic injury. In our study, the fact that differences in morbidity and surgery between people experiencing homelessness and housed patients did not persist in a balanced matched cohort suggests that differences observed in the unmatched cohort were due to high rates of underlying comorbidities and increased injury severity in people experiencing homelessness.

The present study builds on existing literature of increased hospital resource use among people experiencing homelessness.^32,33,34^ Reasons for increased LOS among people experiencing homelessness are multifactorial. It may be driven in part by low socioeconomic status; patients with social and/or material deprivation have been shown to have longer LOS compared with patients with higher socioeconomic status.^35^ Prior studies have also shown that up to 80% of avoidable hospital days are due to delays in accessing postdischarge care.^36^ People experiencing homelessness may not have caregivers to assist them after discharge and may lack resources to pay for postdischarge care.^37,38^ Length of stay may also be prolonged by concomitant physical health and behavioral health comorbidities.^39^ High proportions of people experiencing homelessness have public health insurance, which has been linked to increased LOS in other studies.^40^ Admitted people experiencing homelessness may also receive health and social service resource referrals, which may prolong LOS.^41^ While this study investigated LOS among traumatically injured people experiencing homelessness, results of increased LOS are likely generalizable to people experiencing homelessness hospitalized for other conditions.

Importantly, this study identifies groups of people experiencing homelessness with disproportionately increased LOS: those 65 years or older and those with minor injuries. The US population of people experiencing homelessness is aging and is developing geriatric conditions at younger ages than housed adults.^42,43,44^ Geriatric patients have been shown to have more complications and longer LOS than younger patients due to frailty and comorbidities.^45^ Older patients also frequently lack familial caregiver support.^35^ These challenges are likely amplified in older unhoused patients, leading to greater disparities. Our findings of increased associations between homelessness and hospital use for patients with minor injury are consistent with those of previous work. In 1 study,^9^ differences in rates of hospital admission between people experiencing homelessness and housed patients were greater for patients with minor injuries than patients with severe injuries. The timing of discharge for patients with minor injury may be more clinically discretionary and therefore more susceptible to prolongation by vulnerabilities such as homelessness. Therefore, there may be greater opportunity to reduce disparities in LOS among people experiencing homelessness with minor injury.

These results have important implications for a broad range of stakeholders invested in improving quality and cost of care. Our findings of increased adjusted LOS among people experiencing homelessness undoubtedly translate to significantly increased costs, and prolonged LOS can lead to decreased bed availability and resources for other patients. Improving the care of high-need, high-cost patients such as those experiencing homelessness is a priority for Centers for Medicare & Medicaid Services.^46^ This work contributes to a growing body of evidence that health-related social drivers such as homelessness can exacerbate health disparities and lead to higher cost for public payer programs.^47^ People experiencing homelessness would benefit from programs to make discharge safe and feasible, and there is increasingly a business case for society to invest in interventions that address social determinants of health.^47,48^ These may comprise initiatives to promote affordable housing, improved ambulatory care access, expanded respite programs to support safe and efficient discharge, and care coordination services.^49^ The results of our study suggest that older people experiencing homelessness and those with minor injuries in particular may benefit most from efforts to reduce LOS.

### Limitations

There are several limitations to this study. Many are inherent to the use of national registry datasets. These data are retrospectively analyzed after being collected for measurement of hospital quality. First, there could be unobserved variable bias. However, rigorous data abstraction constitutes an important strength of such data, particularly for trauma-specific fields such as ISS.^20^ Second, misclassification bias may have been introduced in our use of the TQP’s alternate home residence variable to define our cohort of people experiencing homelessness. This variable is completed when a patient’s residential zip code is unknown. It would not capture patients whose documents list the zip code of a former residence or shelter or patients who are temporarily unhoused. The TQP likely underestimates the prevalence of people experiencing homelessness. However, demographic and injury characteristics of the cohort experiencing homelessness are similar to those in other studies, suggesting face validity of the cohort.^33,50^ Third, TQP does not contain income data, a known factor associated with LOS in patients with traumatic injury.^35^ We addressed this by including insurance as a matching criteria in our propensity score model. Insurance status is a proxy for income in US-based studies, as eligibility is largely based on modified adjusted gross income.^51,52^

## Conclusions

This findings of this cohort study suggest that people experiencing homelessness in North America had significantly increased adjusted LOS after hospitalization for traumatic injury compared with housed patients. Older patients and those with minor injuries had disproportionately greater increased adjusted LOS. These findings have significant implications for quality and costs of care for people experiencing homelessness and underscore potential opportunities to reduce disparities in trauma outcomes and improve hospital resource use among those with injuries.

## Notes

Supplement 1. **eTable 1.** Characteristics of 8665 Matched Pairs of People Experiencing Homelessness and Housed Patients in the Trauma Quality Programs, 2017 to 2018 **eTable 2.** Unadjusted Morbidity, Surgery, and Length of Stay Among 8665 Matched Pairs of People Experiencing Homelessness and Housed Patients in the Trauma Quality Programs, 2017 to 2018 **eTable 3.** Multivariable Model for Length of Stay Greater Than 30 Days in 8665 Matched Pairs of People Experiencing Homelessness and Housed Patients in the Trauma Quality Programs, 2017 to 2018

Supplement 2.
Data Sharing Statement

## References

[1] US Department of Housing and Urban Development. The 2020 Annual Homeless Assessment Report (AHAR) to Congress. January 2021. Accessed August 26, 2022. https://www.huduser.gov/portal/sites/default/files/pdf/2020-AHAR-Part-1.pdf

[2] Castrucci BC, Auerbach J. Meeting individual social needs falls short of addressing social determinants of health. *Health Affairs*. January 16, 2019. Accessed May 5, 2023. https://www.healthaffairs.org/content/forefront/meeting-individual-social-needs-falls-short-addressing-social-determinants-health

[3] Abel MK, Lin JA, Wick EC. How can we improve surgical care of patients who are homeless? JAMA Surg. 2022;157(9):846-847. doi:10.1001/jamasurg.2022.2586 35793117

[4] Fazel S, Geddes JR, Kushel M. The health of homeless people in high-income countries: descriptive epidemiology, health consequences, and clinical and policy recommendations. Lancet. 2014;384(9953):1529-1540. doi:10.1016/S0140-6736(14)61132-6 25390578 PMC4520328

[5] Sun S, Irestig R, Burström B, Beijer U, Burström K. Health-related quality of life (EQ-5D) among homeless persons compared to a general population sample in Stockholm County, 2006. Scand J Public Health. 2012;40(2):115-125. doi:10.1177/1403494811435493 22327187

[6] Ku BS, Fields JM, Santana A, Wasserman D, Borman L, Scott KC. The urban homeless: super-users of the emergency department. Popul Health Manag. 2014;17(6):366-371. doi:10.1089/pop.2013.0118 24865472

[7] Baggett TP, Liauw SS, Hwang SW. Cardiovascular disease and homelessness. J Am Coll Cardiol. 2018;71(22):2585-2597. doi:10.1016/j.jacc.2018.02.077 29852981

[8] White BM, Newman SD. Access to primary care services among the homeless: a synthesis of the literature using the equity of access to medical care framework. J Prim Care Community Health. 2015;6(2):77-87. doi:10.1177/2150131914556122 25389222

[9] Silver CM, Thomas AC, Reddy S, . Injury patterns and hospital admission after trauma among people experiencing homelessness. JAMA Netw Open. 2023;6(6):e2320862. doi:10.1001/jamanetworkopen.2023.20862 37382955 PMC10311388

[10] Salit SA, Kuhn EM, Hartz AJ, Vu JM, Mosso AL. Hospitalization costs associated with homelessness in New York City. N Engl J Med. 1998;338(24):1734-1740. doi:10.1056/NEJM199806113382406 9624194

[11] Rollings KA, Kunnath N, Ryus CR, Janke AT, Ibrahim AM. Association of coded housing instability and hospitalization in the US. JAMA Netw Open. 2022;5(11):e2241951. doi:10.1001/jamanetworkopen.2022.41951 36374498 PMC9664259

[12] Brasel KJ, Lim HJ, Nirula R, Weigelt JA. Length of stay: an appropriate quality measure? Arch Surg. 2007;142(5):461-465. doi:10.1001/archsurg.142.5.461 17515488

[13] Hwang SW, Weaver J, Aubry T, Hoch JS. Hospital costs and length of stay among homeless patients admitted to medical, surgical, and psychiatric services. Med Care. 2011;49(4):350-354. doi:10.1097/MLR.0b013e318206c50d 21368678

[14] Buttigieg SC, Abela L, Pace A. Variables affecting hospital length of stay: a scoping review. J Health Organ Manag. 2018;32(3):463-493. doi:10.1108/JHOM-10-2017-0275 29771210

[15] American College of Surgeons. National Trauma Data Standard (NTDS). Accessed October 12, 2022. https://www.facs.org/quality-programs/trauma/quality/national-trauma-data-bank/national-trauma-data-standard/

[16] Ingraham AM, Xiong W, Hemmila MR, . The attributable mortality and length of stay of trauma-related complications: a matched cohort study. Ann Surg. 2010;252(2):358-362. doi:10.1097/SLA.0b013e3181e623bf 20622658

[17] Ingram ME, Nagalla M, Shan Y, . Sex-based disparities in timeliness of trauma care and discharge disposition. JAMA Surg. 2022;157(7):609-616. doi:10.1001/jamasurg.2022.1550 35583876 PMC9118066

[18] American College of Surgeons. National Surgical Quality Improvement Program. Accessed September 14, 2022. https://www.facs.org/quality-programs/data-and-registries/acs-nsqip/

[19] Agency for Healthcare Research and Quality. Patient safety indicators technical specifications (pdf format)—version v2023. August 2023. Accessed September 5, 2023. https://qualityindicators.ahrq.gov/measures/PSI_TechSpec

[20] Nathens AB, Cryer HG, Fildes J. The American College of Surgeons Trauma Quality Improvement Program. Surg Clin North Am. 2012;92(2):441-454, x-xi. doi:10.1016/j.suc.2012.01.003 22414421

[21] Chesley CF, Chowdhury M, Small DS, . Racial disparities in length of stay among severely ill patients presenting with sepsis and acute respiratory failure. JAMA Netw Open. 2023;6(5):e239739. doi:10.1001/jamanetworkopen.2023.9739 37155170 PMC10167564

[22] Stagg V. ELIXHAUSER: Stata module to calculate Elixhauser index of comorbidity. September 16, 2015. Accessed November 7, 2022. https://econpapers.repec.org/software/bocbocode/s458077.htm

[23] Van Ditshuizen JC, Sewalt CA, Palmer CS, Van Lieshout EMM, Verhofstad MHJ, Den Hartog D; Dutch Trauma Registry Southwest. The definition of major trauma using different revisions of the abbreviated injury scale. Scand J Trauma Resusc Emerg Med. 2021;29(1):71. doi:10.1186/s13049-021-00873-7 34044857 PMC8162011

[24] Mehta R, Chinthapalli K. Glasgow Coma Scale explained. BMJ. 2019;365:l1296. doi:10.1136/bmj.l1296 31048343

[25] Centers for Disease Control and Prevention. *ICD-10* framework: injury mortality diagnosis matrix. Updated November 6, 2015. Accessed November 5, 2022. https://www.cdc.gov/nchs/injury/ice/injury_matrix10.htm#:~:text=The%20ICD%2D10%20Injury%20Mortality,region%20and%20nature%20of%20injury

[26] Tsai J, Mehta K, Mongtomery AE, Elbogen E, Hooshyar D. Changing demography of homeless adult populations. Perspect Public Health. 2021;141(3):177-184. doi:10.1177/1757913920919796 32476585

[27] Haukoos JS, Lewis RJ. The propensity score. JAMA. 2015;314(15):1637-1638. doi:10.1001/jama.2015.13480 26501539 PMC4866501

[28] Austin PC. A comparison of 12 algorithms for matching on the propensity score. Stat Med. 2014;33(6):1057-1069. doi:10.1002/sim.6004 24123228 PMC4285163

[29] Silber JH, Rosenbaum PR, Clark AS, . Characteristics associated with differences in survival among Black and White women with breast cancer. JAMA. 2013;310(4):389-397. doi:10.1001/jama.2013.8272 23917289

[30] Cawley C, Kanzaria HK, Zevin B, Doran KM, Kushel M, Raven MC. Mortality among people experiencing homelessness in San Francisco during the COVID-19 pandemic. JAMA Netw Open. 2022;5(3):e221870. doi:10.1001/jamanetworkopen.2022.1870 35267030 PMC8914573

[31] Decker HC, Kanzaria HK, Evans J, Pierce L, Wick EC. Association of housing status with types of operations and postoperative health care utilization. Ann Surg. 2023;278(6):883-889. doi:10.1097/SLA.0000000000005917 37232943

[32] Schaffer KB, Wang J, Nasrallah FS, . Disparities in triage and management of the homeless and the elderly trauma patient. Inj Epidemiol. 2020;7(1):39. doi:10.1186/s40621-020-00262-1 32654664 PMC7358191

[33] Miller JP, O’ Reilly GM, Mackelprang JL, Mitra B. Trauma in adults experiencing homelessness. Injury. 2020;51(4):897-905. doi:10.1016/j.injury.2020.02.086 32147144

[34] Ferrada P, Anand RJ, Aboutanos M. The uninsured, the homeless, and the undocumented immigrant trauma patient: revealing health-care disparity at a level 1 trauma center. Am Surg. 2016;82(1):E1-E2. doi:10.1177/000313481608200101 26802837

[35] Moore L, Cisse B, Batomen Kuimi BL, . Impact of socio-economic status on hospital length of stay following injury: a multicenter cohort study. BMC Health Serv Res. 2015;15:285. doi:10.1186/s12913-015-0949-2 26204932 PMC4513757

[36] Majeed MU, Williams DT, Pollock R, . Delay in discharge and its impact on unnecessary hospital bed occupancy. BMC Health Serv Res. 2012;12(1):410. doi:10.1186/1472-6963-12-410 23167656 PMC3511236

[37] Wong AW, Gan WQ, Burns J, Sin DD, van Eeden SF. Acute exacerbation of chronic obstructive pulmonary disease: influence of social factors in determining length of hospital stay and readmission rates. Can Respir J. 2008;15(7):361-364. doi:10.1155/2008/569496 18949105 PMC2679571

[38] Perelman J, Closon MC. Impact of socioeconomic factors on in-patient length of stay and their consequences in per case hospital payment systems. J Health Serv Res Policy. 2011;16(4):197-202. doi:10.1258/jhsrp.2011.010047 21965425

[39] Lygrisse KA, Singh V, Oakley CT, . Effect of documented and undocumented psychiatric conditions on length of stay and discharge destination after total knee arthroplasty. Arch Orthop Trauma Surg. 2023;143(3):1571-1578. doi:10.1007/s00402-022-04415-3 35318485

[40] Englum BR, Hui X, Zogg CK, . Association between insurance status and hospital length of stay following trauma. Am Surg. 2016;82(3):281-288. doi:10.1177/000313481608200324 27099067 PMC5142530

[41] Doran KM, Boyer AP, Raven MC. Health care for people experiencing homelessness—what outcomes matter? JAMA Netw Open. 2021;4(3):e213837. doi:10.1001/jamanetworkopen.2021.3837 33764419

[42] Culhane DP, Metraux S, Byrne T, Stino M, Bainbridge J. The age structure of contemporary homelessness: evidence and implications for public policy. Anal Soc Issues Public Policy. 2013;13(1):228-244. doi:10.1111/asap.12004

[43] Brown RT, Kiely DK, Bharel M, Mitchell SL. Factors associated with geriatric syndromes in older homeless adults. J Health Care Poor Underserved. 2013;24(2):456-468. doi:10.1353/hpu.2013.0077 23728022 PMC3671483

[44] Suh K, Beck J, Katzman W, Allen DD. Homelessness and rates of physical dysfunctions characteristic of premature geriatric syndromes: systematic review and meta-analysis. Physiother Theory Pract. 2022;38(7):858-867. doi:10.1080/09593985.2020.1809045 32835565

[45] CDC/NCHS National Hospital Discharge Survey. Number, percent distribution, rate, days of care with average length of stay, and standard error of discharges from short-stay hospitals, by sex and age: United States, 2010. 2021. Accessed March 2, 2023. https://www.cdc.gov/nchs/data/nhds/2average/2010ave2_ratesexage.pdf

[46] Blumenthal D, Abrams MK. Tailoring complex care management for high-need, high-cost patients. JAMA. 2016;316(16):1657-1658. doi:10.1001/jama.2016.12388 27669168

[47] Lipson D. Medicaid’s role in improving the social determinants of health: opportunities for states. June 2017. Accessed March 3, 2023. https://www.nasi.org/wp-content/uploads/2017/06/Opportunities-for-States_web.pdf

[48] Costello A. Opportunies in Medicaid and CHIP to address social determinants of health (SDOH). January 7, 2021. Accessed March 3, 2023. https://www.medicaid.gov/sites/default/files/2022-01/sho21001_0.pdf

[49] Doran KM, Ragins KT, Gross CP, Zerger S. Medical respite programs for homeless patients: a systematic review. J Health Care Poor Underserved. 2013;24(2):499-524. doi:10.1353/hpu.2013.0053 23728025

[50] Kramer CB, Gibran NS, Heimbach DM, Rivara FP, Klein MB. Assault and substance abuse characterize burn injuries in homeless patients. J Burn Care Res. 2008;29(3):461-467. doi:10.1097/BCR.0b013e31817112b0 18388565 PMC3042353

[51] Medicaid.gov. Medicaid eligibility. 2022. Accessed August 22, 2022. https://www.medicaid.gov/medicaid/eligibility/index.html

[52] Casey JA, Pollak J, Glymour MM, Mayeda ER, Hirsch AG, Schwartz BS. Measures of SES for electronic health record–based research. Am J Prev Med. 2018;54(3):430-439. doi:10.1016/j.amepre.2017.10.004 29241724 PMC5818301

