# Supplementary material for: Morbidity and Length of Stay After Injury Among People Experiencing Homelessness in North America
Source: JAMA Netw Open. 2024 Feb 28;7(2):e240795. doi: 10.1001/jamanetworkopen.2024.0795 (PMC10902734; doi:10.1001/jamanetworkopen.2024.0795)
Supplement: Supplement 1. — eTable 1. Characteristics of 8665 Matched Pairs of People Experiencing Homelessness and Housed Patients in the Trauma Quality Programs, 2017 to 2018 eTable 2. Unadjusted Morbidity, Surgery, and Length of Stay Among 8665 Matched Pairs of People Experiencing Homelessness and Housed Patients in the Trauma Quality Programs, 2017 to 2018 eTable 3. Multivariable Model for Length of Stay Greater Than 30 Days in 8665 Matched Pairs of People Experiencing Homelessness and Housed Patients in the Trauma Quality Programs, 2017 to 2018 [file jamanetwopen-e240795-s001.pdf]

# Supplementary Online Content

Silver CM, Thomas AC, Reddy S, et al. Morbidity and length of stay after injury among people experiencing homelessness in North America. *JAMA Netw Open*. 2024;7(3):e240795. doi:10.1001/jamanetworkopen.2024.0795

**eTable 1.** Characteristics of 8665 Matched Pairs of People Experiencing Homelessness and Housed Patients in the Trauma Quality Programs, 2017 to 2018

**eTable 2.** Unadjusted Morbidity, Surgery, and Length of Stay Among 8665 Matched Pairs of People Experiencing Homelessness and Housed Patients in the Trauma Quality Programs, 2017 to 2018

**eTable 3.** Multivariable Model for Length of Stay Greater Than 30 Days in 8665 Matched Pairs of People Experiencing Homelessness and Housed Patients in the Trauma Quality Programs, 2017 to 2018

This supplementary material has been provided by the authors to give readers additional information about their work.

© 2024 Silver CM et al. *JAMA Network Open*.

**eTable 1.** Characteristics of 8665 Matched Pairs of People Experiencing Homelessness and Housed Patients in the Trauma Quality Programs, 2017 to 2018

| Characteristics | **People Experienceing Homelessness** N=8,665 (50.0%) | **Housed Patients** N=8,665 (50.0%) | ***Standardized Difference***[a] |
|---|---|---|---|
| Sex | | | |
| Male | 7,312 (84.4) | 7,361 (85.0) | 0.01[d] |
| Female | 1,353 (15.6) | 1,304 (15.0) | |
| Age (years) | | | |
| 18-35 | 2,324 (26.9) | 3,488 (41.0) | 0.41 |
| 36-50 | 2,695 (31.2) | 2,152 (25.3) | |
| 51-64 | 3,069 (35.5) | 1,888 (22.2) | |
| $\geq$ 65 | 559 (6.5) | 972 (11.4) | |
| Race/Ethnicity | | | |
| Non-Hispanic White | 4,375 (51.8) | 4,096 (48.3) | 0.15 |
| Non-Hispanic Black | 1,816 (21.5) | 1,578 (18.6) | |
| Hispanic | 887 (10.5) | 1,003 (11.8) | |
| Other | 1,369 (16.2) | 1,805 (21.3) | |
| Insurance | | | |
| Private | 1,022 (11.8) | 1,156 (13.3) | 0.07[d] |
| Uninsured | 1,895 (21.9) | 1,803 (20.8) | |
| Medicaid | 4,346 (50.2) | 4,142 (47.8) | |
| Medicare | 874 (10.1) | 976 (11.3) | |
| Other | 528 (6.1) | 588 (6.8) | |
| Comorbidities | | | |
| Any physical comorbidity[b] | 3,130 (36.1) | 3,038 (35.1) | 0.02[d] |
| Any behavioral comorbidity[c] | 1,973 (22.8) | 1,886 (21.8) | 0.02[d] |
| **Injury Characteristics** | | | |
| Injury Mechanism Type | | | |
| Blunt | 6,599 (76.2) | 6,619 (76.4) | 0.01[d] |
| Penetrating | 1,488 (17.2) | 1,498 (17.3) | |
| Unknown/Other | 578 (6.7) | 548 (6.3) | |
| Injury Body Region | | | |
| Head or Neck | 3,183 (36.7) | 3,195 (36.9) | 0.02[d] |
| Spine | 560 (6.5) | 560 (6.5) | |
| Torso | 3,571 (41,2) | 3,558 (41.1) | |
| Extremity | 1,299 (15.0) | 1,311 (15.1) | |
| Injury Severity Score | | | |
| Mild injury: 1-8 | 4,039 (46.7) | 3,916 (45.2) | 0.03 |
| Moderate injury: 9-12 | 2,859 (33.0) | 2,971 (34.3) | |
| Severe injury: $\geq$16 | 1,756 (20.3) | 1,769 (20.4) | |
| Initial GCS Score | | | |
| 3-8 | 557 (6.4) | 572 (6.6) | 0.06[d] |
| 9-12 | 497 (5.7) | 384 (4.4) | |
| 13-15 | 7,611 (87.8) | 7,709 (89.0) | |

*Abbreviation: GCS Glasgow Coma Scale*

[a] Standardized differences are equal to the absolute value of the difference in proportions divided by the standard error. It is considered an indicator of effect size: >0.20 indicates small, >0.50 medium, and $\geq$0.80 large effect size

[b] Medical comorbidities include heart disease, hypertension, chronic obstructive pulmonary disease, chronic kidney disease, diabetes, malignancy, or liver disease

[c] Behavioral comorbidities include schizophrenia, bipolar disorder, major depressive disorder, social anxiety disorder, posttraumatic stress disorder, and antisocial personality disorder

[d] Characteristics on which patients were propensity score matched

© 2024 Silver CM et al. *JAMA Network Open*.

**eTable 2.** Unadjusted Morbidity, Surgery, and Length of Stay Among 8665 Matched Pairs of Patients Experiencing Homelessness and Housed Patients in the Trauma Quality Programs, 2017 to 2018

| **Hospital Event** | **People Experienceing Homelessness** N=8,665 (50.0%) | **Housed Patients** N=8,665 (50.0%) | ***P value***[a] |
|---|---|---|---|
| Morbidity | | | |
| Acute Kidney Injury | 36 (0.4) | 40 (0.5) | 0.65 |
| Acute Respiratory Distress Syndrome | 18 (0.2) | 30 (0.3) | 0.08 |
| Cardiac Arrest with CPR | 26 (0.3) | 28 (0.3) | 0.78 |
| Venous Thromboembolism | 27 (0.3) | 41 (0.5) | 0.09 |
| Myocardial Infarction | 5 (0.1) | 3 (<0.1) | 0.48 |
| Severe Sepsis | 27 (0.3) | 28 (0.3) | 0.89 |
| Surgical Site Infection | 25 (0.3) | 11 (0.1) | 0.02 |
| Unplanned Intubation | 109 (1.3) | 78 (0.9) | 0.02 |
| Any Morbidity | 214 (2.5) | 199 (2.3) | 0.45 |
| Hemorrhage Control Surgery | 285 (3.3) | 299 (3.4) | 0.53 |
| ICU Admission | 2,271 (26.2) | 2,217 (25.6) | 0.30 |
| LOS, days (continuous), median (IQR) | 5 (3-10) | 4 (2-8) | <0.001 |
| LOS, days (continuous), mean (SD) | 9.5 (15.4) | 7.4 (11.3) | <0.001 |
| LOS >30 days | 478 (5.5) | 327 (3.8) | <0.001 |

*Abbreviations: CPR cardiopulmonary resuscitation; ICU intensive care unit; LOS length of stay; IQR interquartile range; SD standard deviation*

[a] *P* values derived from McNemar's tests (categorical variables) and Wilcoxon signed-rank test (continuous variables).

© 2024 Silver CM et al. *JAMA Network Open*.

**eTable 3.** Multivariable Model for Length of Stay Greater Than 30 Days in 8665 Matched Pairs of People Experiencing Homelessness and Housed Patients in the Trauma Quality Programs, 2017 to 2018

| Characteristic | OR (95% CI) | *P value*[a] |
|---|---|---|
| Housing Status | | |
| Housed Patients | Reference | |
| People Experiencing Homelessness | 1.59 (1.33-1.89) | <0.001 |
| Age | | |
| 18-35 | Reference | |
| 36-50 | 1.21 (0.96-1.54) | 0.10 |
| 51-64 | 1.66 (1.32-2.08) | <0.001 |
| >= 65 | 1.85 (1.35-2.55) | <0.001 |
| Injury Severity Score | | |
| 1-8 | Reference | |
| 9-15 | 2.54 (1.93-3.33) | <0.001 |
| >=16 | 9.52 (7.21-12.58) | <0.001 |
| Any Morbidity | | |
| No | Reference | |
| Yes | 12.49 (9.03-17.27) | <0.001 |
| Hemorrhage Control Surgery | | |
| No | Reference | |
| Yes | 2.95 (2.16-4.03) | <0.001 |
| ICU Admission | | |
| No | Reference | |
| Yes | 2.35 (1.94-2.84) | <0.001 |

*Abbreviations: OR odds ratio; CI confidence interval; ICU intensive care unit*

[a] *P* values are estimated from hierarchical multivariable logistic regression models allowing for random effects at the pair level and at the hospital level.

© 2024 Silver CM et al. *JAMA Network Open*.
