# Supplementary material for: Morbidity and Length of Stay After Injury Among People Experiencing Homelessness in North America
Source: JAMA Netw Open. 2024 Feb 28;7(2):e240795. doi: 10.1001/jamanetworkopen.2024.0795 (PMC10902734; doi:10.1001/jamanetworkopen.2024.0795)
Supplement: Supplement 2. — Data Sharing Statement [file jamanetwopen-e240795-s002.pdf]

Data Sharing Statement

Silver. Morbidity and Length of Stay After Injury Among People Experiencing Homelessness in North America. *JAMA Netw Open*. Published February 28, 2024.
doi:10.1001/jamanetworkopen.2024.0795

Data
Data available: No
